# Endogenous hyperinsulinemic hypoglycemia: case series and literature review

**DOI:** 10.1007/s12020-022-03268-5

**Published:** 2022-12-02

**Authors:** Chenyang Zhang, Hui Zhang, Wen Huang

**Affiliations:** Department of General Practice, The First Affiliated Hospital of Shandong First Medical University & Shandong Provincial Qianfoshan Hospital, Jinan, Shandong province 250014 China

**Keywords:** Endogenous hyperinsulinemic hypoglycemia, Insulinoma, Type B insulin resistance syndrome, Insulin autoimmune syndrome

## Abstract

**Purpose:**

Endogenous hyperinsulinemic hypoglycemia (EHH) is an uncommon disease characterized by inappropriately high plasma insulin levels despite low plasma glucose levels. Some rare etiologies can lead to EHH. Correct diagnosis is a prerequisite for treatment. Hence, although challenging, it is crucial for patients with EHH to identify the different causes.

**Methods:**

We describe a case series of three patients, all of whom had obvious hypoglycemic symptoms and extraordinary hyperinsulinemia. Their plasma glucose, insulin, and C-peptide levels were tested simultaneously when hypoglycemia occurred. Moreover, other biochemical indices and relevant antibody levels were measured and imaging examinations were conducted.

**Results:**

According to their medical history, physical examination, laboratory results, and imaging findings, the three patients were diagnosed with insulinoma, type B insulin resistance syndrome, and insulin autoimmune syndrome. After precise treatments, hypoglycemia was ultimately eliminated.

**Conclusion:**

Although these diseases have similar symptoms and biochemical abnormalities, the treatment and prognosis are different. The case series presented here highlights the challenges in the differential diagnosis of EHH. An accurate diagnosis is necessary for hypoglycemia treatment.

## Introduction

Hypoglycemia is common in the clinical setting except for that caused by endogenous hyperinsulinemia, which is very rare. Endogenous hyperinsulinemic hypoglycemia (EHH), which can be caused by different etiologies, is a condition in which insulin levels are inappropriately high in the presence of low plasma glucose levels [1]. Despite the similarity in symptoms and biochemical abnormalities, the therapeutic approaches and prognosis of these diseases are different; thus, making a proper diagnosis is critical. Here we present a case series of three EHH patients with different etiologies. By analyzing and discussing the features and causes of EHH, we hope to provide a useful reference for clinical work.

## Case reports

### Case 1

**Fig. 1:**
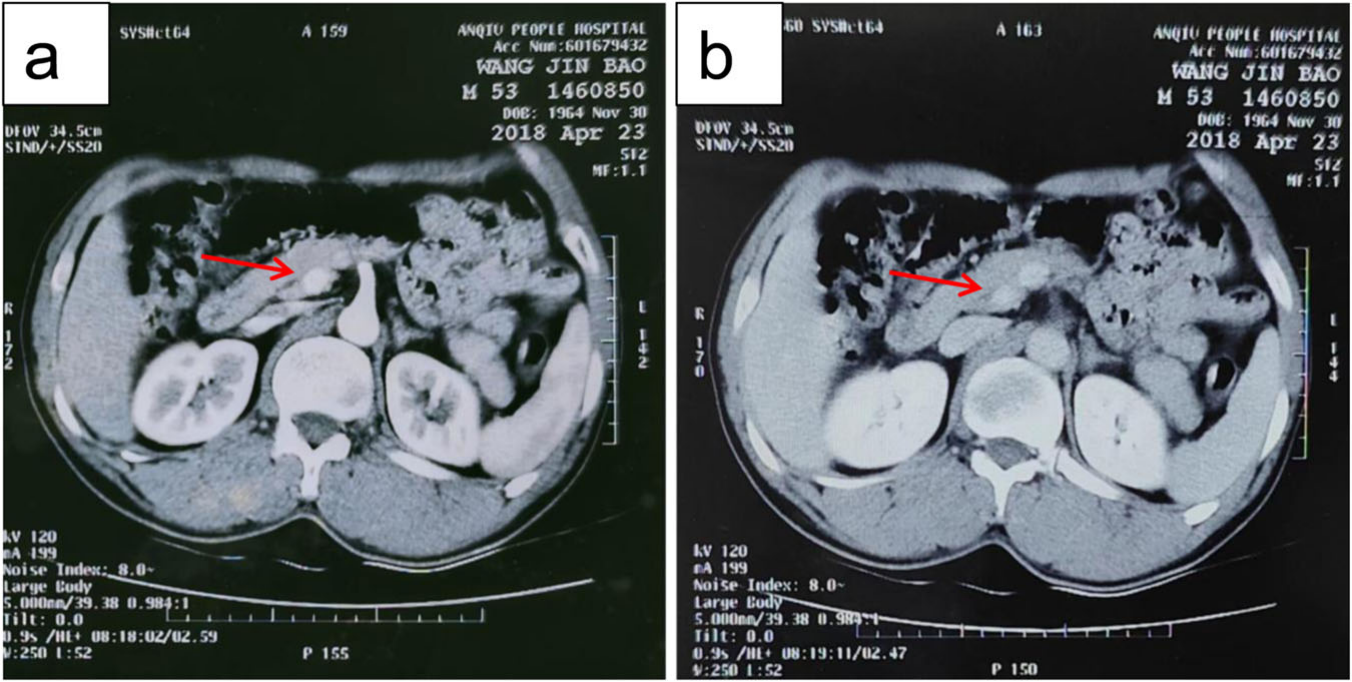
Abdomen enhanced CT scan of case 1. **a**, **b** Abdomen enhanced CT revealed a round, well-circumscribed, and arterialized lesion at the tail of the pancreas (arrow)

A 79-year-old Chinese male presented to the emergency department with sudden unconsciousness. He was found to have severe hypoglycemia with 1.01 mmol/L blood glucose. After an intravenous glucose injection, the patient regained consciousness. He complained of having had repeated dizziness and palpitation for the past 10 months. He had no history of bariatric or gastric surgery and no family history of diabetic mellitus. Physical examination was unremarkable, and his hypoglycemic symptoms repeatedly occurred during hospitalization. The blood glucose level was 2.42 mmol/L, accompanied by an inappropriately high serum insulin level of 117.8 µIU/ml and a C-peptide level of 8.87 ng/ml. The insulin release index (serum insulin divided by blood glucose) was 2.70 (normal values are <0.3). His laboratory results on admission are shown in Table 1. His thyroid function and growth hormone, insulin-like growth factor-1, and cortisol levels were within their normal ranges. Abdomen-enhanced computerized tomography (CT) scans showed a round mass at the tail of the pancreas, approximately 0.8 cm in diameter (Fig. 1). He subsequently underwent laparoscopic partial pancreatectomy. Histopathology revealed that the lesion was a neuroendocrine tumor, positive for insulin. The patient was diagnosed with insulinoma. His blood glucose levels returned to normal and hypoglycemia did not recur after the operation.

**Table 1:** Laboratory examination results in Case 1

Parameters	Results	Reference range
white blood cells (×10^9^/L)	5.54	3.5–9.5
red blood cells (×10^12^/L)	4.70	3.8–5.1
platelets (×10^9^/L)	190	125–350
albumin (g/L)	45.6	40–55
serum creatinine (umol/L)	91.0	59–104
sodium (mmol/L)	142.0	137–147
potassium (mmol/L)	3.76	3.5–5.3
total bilirubin (umol/L)	10.8	5.0–24.0
aspartate aminotransferase (U/L)	15.0	<40
alanine aminotransferase (U/L)	11.7	<50
fast blood glucose (mmol/L)	1.80	3.89–6.11
C-peptide (ng/mL)	2.86	1.1–4.4
insulin (µIU/ml)	14.37	2.6–24.9
HbA1c (%)	4.7	4–6
grow hormone (ng/ml)	1.86	0.06–5.0
IGF-1 (μg/mL)	140.2	60–350
cortisol 8 am (nmol/L)	294.2	172–497
ACTH 8 am (pg/ml)	29.94	5–60
free T3 (pmol/L)	4.32	3.1–6.8
free T4 (pmol/L)	12.48	12–22
TSH (μIU/mL)	1.99	0.27–4.2
anti-TPO antibody (IU/mL)	10.56	0–34
anti-TG antibody (IU/mL)	13.80	0–115

### Case 2

**Fig. 2:**
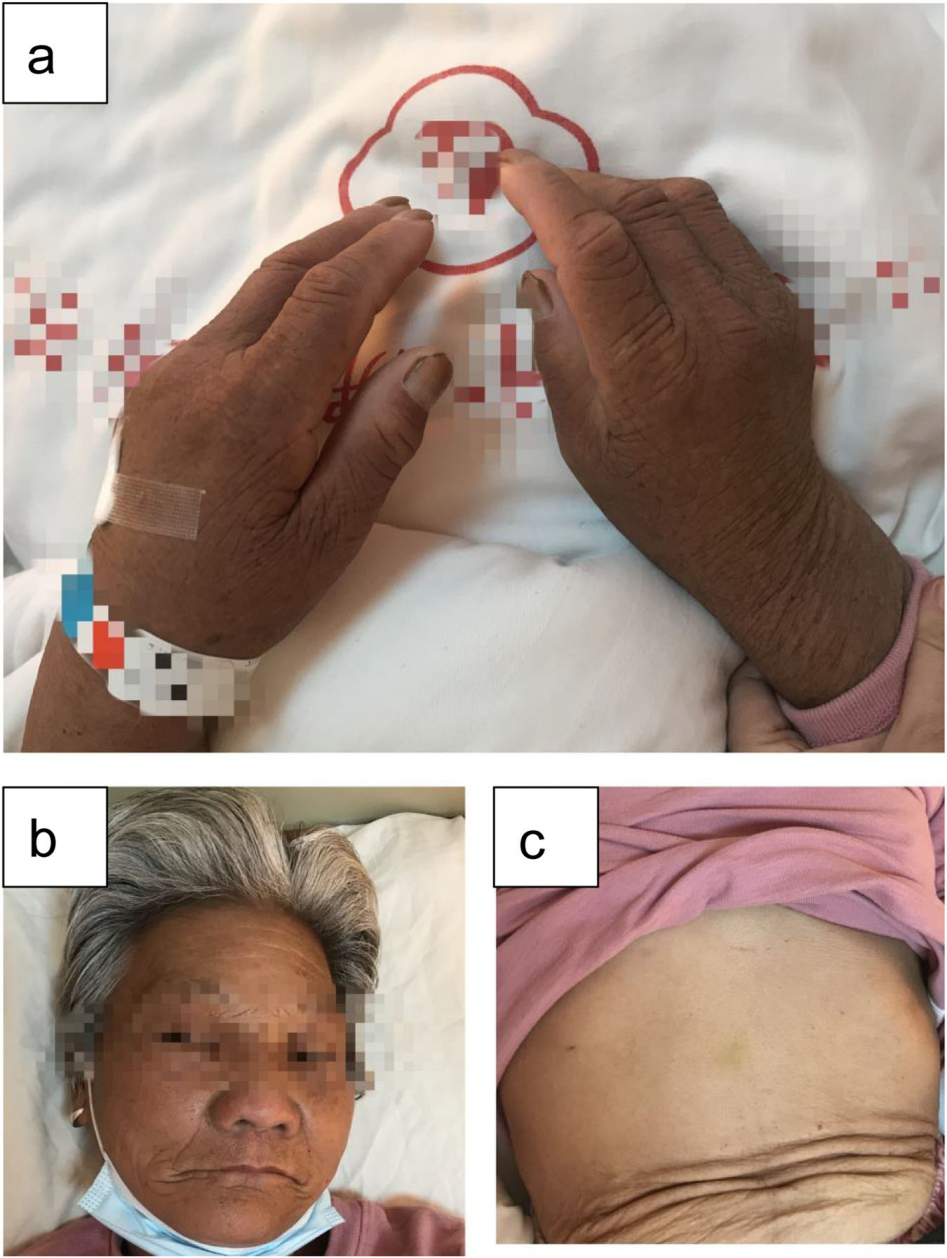
Skin appearance of case 2. **a**–**c** Thickened, hyperpigmented skin lesions were observed on the hands and face compared to the abdomen

A 72-year-old Chinese female suddenly presented with sweating and subsequent loss of consciousness. She automatically regained consciousness after half an hour. She had been having the same experience twice for the past 5 months. The patient was taken to the local hospital, and her blood glucose level was only 2.10 mmol/L. During hospitalization, she underwent an oral glucose tolerance test (OGTT) (Table 2). She was diagnosed with diabetes mellitus and treated with insulin, but she developed frequent hypoglycemia and had to stop taking insulin. The patient was transferred to our hospital for a definitive diagnosis. She had no history of hyperthyroidism or sulfhydryl medication. She had no history of autoimmune disease. Her BMI was 21.29 kg/m^2^, and she had dark and rough skin on her face, neck, and hands (Fig. 2). Blood glucose monitoring revealed obvious blood glucose fluctuations (2.7–17.37 mmol/L) (Table 2). When her serum glucose was 2.69 mmol/L, the synchronous serum insulin level was 326.20 μIU/mL, with normal C-peptide levels. Despite recurrent hypoglycemia, her hemoglobin A1c (HbA1c) level remained high (10.8%). Her serum insulin autoantibody (IAA) level was weakly elevated. Table 2 presents her laboratory results on admission. Her thyroid function and growth hormone, insulin-like growth factor-1, and cortisol levels were within their normal ranges. Her liver function was slightly impaired, with elevated γ-glutamyl transpeptidase and alkaline phosphatase levels. Her testosterone level was elevated. The anti-nuclear antibody titer was high (1:320), with a cytoplasmic particle pattern. Moreover, she was positive for anti-SSA antibody and anti-AMA-M2 antibody. An ophthalmic examination revealed xerophthalmia. Chest and abdomen CT scans revealed no abnormalities. Therefore, the clinical characteristics of the patient were both severe hypoglycemia and significant hyperglycemia, hyperinsulinemia, extreme insulin resistance, hyperandrogenism, and autoimmune disease. After ruling out other causes of hypoglycemia, type B insulin resistance syndrome (TBIRS) was considered according to the current clinical characteristics and laboratory results, although insulin receptor antibody was not detected due to technical limitations. She had coexistent Sjogren’s syndrome and primary biliary cirrhosis. We treated her with methylprednisolone (20 mg BID) for immunomodulation, insulin aspart (8 units), and metformin (500 mg) before meals to control hyperglycemia. Her blood glucose levels were stabilized between 5.8 and 12.8 mmol/L. Methylprednisolone was eventually reduced to 4 mg BID. The patient experienced no hypoglycemic attacks during her outpatient follow-up.

**Table 2:** Laboratory examination results in Case 2

Parameters	Results					Reference range
OGTT results (local hospital)	0 h	1 h	2 h	3 h		
blood glucose (mmol/L)	13.05	12.98	18.37	18.95		3.89–6.11
serum Insulin (pmol/L)	2083	2599	4277	4305		21.53–121.98
C peptide (ng/ml)	2.47	2.26	3.72	3.74		0.78–5.19
Blood glucose monitoring on the different times after admission						
blood glucose (mmol/L)	2.14	2.66	2.69	17.37	12.45	3.89–6.11
serum Insulin (μIU/mL)	285.40	351.50	326.20	839.50	832.10	2.60–24.90
C peptide (ng/mL)	1.76	2.10	2.16	10.48	9.46	1.10–4.40
IAA (IU/mL)	47.54	44.69	47.65	52.74	34.32	0.41–20
Laboratory test results on admission						
white blood cells (× 10^9^/L)	3.02					3.5–9.5
red blood cells (× 10^12^/L)	3.56					3.8–5.1
platelets (× 10^9^/L)	131					125–350
albumin (g/L)	33					40–55
serum creatinine (umol/L)	60					45–84
sodium (mmol/L)	141					137–147
potassium (mmol/L)	3.56					3.5–5.3
total bilirubin (umol/L)	5.5					5.0–24.0
aspartate aminotransferase (U/L)	26.5					< 35
alanine aminotransferase (U/L)	22.20					< 40
alkaline phosphatase (U/L)	154					45–125
γ-glutamyl transpeptidase (U/L)	54					<45
HbA1c (%)	10.8					4–6
grow hormone (ng/ml)	3.88					0.06–5.0
IGF-1 (μg/mL)	45.9					60–350
cortisol 8 am (nmol/L)	329.8					172–497
ACTH 8 am (pg/ml)	53.98					5–60
testosterone (ng/mL)	2.89					0.06–0.82
free T3 (pmol/L)	3.69					3.1–6.8
free T4 (pmol/L)	14.18					12–22
TSH (μIU/mL)	1.39					0.27–4.2
anti-TPO antibody (IU/mL)	14.56					0–34
anti-TG antibody (IU/mL)	18.13					0–115
anti-nuclear antibody	1:320					negative
anti-SSA antibody	positive					negative
anti-SSB antibody	negative					negative
anti-dsDNA antibody	negative					negative
anti-AMA-M2 antibody	positive					negative

### Case 3

A 68-year-old male was admitted to our hospital with repeated episodes of dizziness, palpitations, and fatigue for the past 1 month. He was diagnosed with type 2 diabetes mellitus in 2017. His blood glucose had been well controlled through diet control and metformin (500 mg TID). Three months ago, he was treated with simvastatin due to hyperlipidemia and developed drug-induced liver damage. He discontinued metformin, which was replaced with insulin aspart 30R therapy. Meanwhile, diammonium glycyrrhizinate and glutathione were given to improve liver function. His blood glucose level was stabilized between 6 and 7 mmol/L. One month later, his liver transaminase levels returned to the normal range, but he started to frequently complain of palpitation, hunger sensation, and diaphoresis with low blood glucose levels of 3.0–4.0 mmol/L. Although he stopped insulin, the blood glucose levels remained unstable, fluctuating between 2.8 and 21.6 mmol/L. He had no history of hyperthyroidism. As shown in Table 3, his OGTT demonstrated severe hyperinsulinemia (17470 to 23650 μIU/ml). More importantly, his IAA was strongly positive. Abdominal contrast-enhanced CT revealed that the pancreas and gastrointestinal tract were normal, with no suspicious masses resembling insulinomas. Based on the above results, the patient was diagnosed with insulin autoimmune syndrome (IAS). When the patient was treated with prednisone (5 mg TID) and acarbose (0.5 g TID) for 2 weeks, he did not experience hypoglycemic symptoms and his blood glucose levels were stabilized around 6.5 mmol/L. Three months after being discharged, the patient gradually terminated glucocorticoids. Ten months later, follow-up examinations showed that the initial positive result for insulin autoantibodies turned negative.

**Table 3:** OGTT results in Case 3

Parameters	Results					Reference range
	0 h	0.5 h	1 h	2 h	3 h	
blood glucose (mmol/L)	3.8	9.7	14.2	18.2	9.9	3.89–6.11
serum Insulin (µIU/ml)	20900	23650	21320	20480	17470	2.60–24.90
C peptide (ng/ml)	>10	>10	>10	>10	>10	1.10–4.40

## Discussion

EHH is an uncommon disease characterized by low blood glucose levels due to excessive endogenous insulin. Hypoglycemic episodes can result in palpitations, hunger, diaphoresis, confusion, and even loss of consciousness. EHH diagnosis is based on plasma insulin >3 µIU/ml, plasma C-peptide > 0.6 ng/ml, and concomitant blood glucose <3 mmol/L [2, 3]. EHH could be caused by various etiologies, such as insulinoma, TBIRS, and IAS. Accurate diagnosis is crucial for the effective treatment of EHH. In this article, we provide a case series and review the related literature to discuss the clinical features and causes of EHH.

Insulinoma is an insulin-secreting tumor, which is the most common cause of EHH in adults [4]. The majority of cases are benign, solitary, small, and intrapancreatic [5]. Its clinical manifestations are mainly Whipple’s triad. Although a 72-h starvation induction test is recommended as the biochemical golden standard to diagnose insulinoma [6], the most commonly used approach is opportune blood testing of plasma glucose, insulin, and C-peptide levels during hypoglycemic episodes [7]. The insulin release index, i.e., the ratio of plasma insulin to blood glucose, is an index to judge whether too much endogenous insulin is secreted. Normal insulin release index values are less than 0.3, but in insulinoma patients, this value can reach more than 0.4 or even more than 1.0 [8]. Localization studies are carried out once there is convincing biochemical evidence to support insulinoma. CT and MRI of the abdomen are the most commonly used imaging modalities. If these tests do not identify a tumor, endoscopic ultrasound or selective angiography is subsequently performed [5]. The therapy of first choice is surgical resection. In the current study, case 1 presented Whipple’s triad accompanied by moderately increased insulin and C-peptide levels when hypoglycemia occurred. His insulin release index was 2.70. Further, abdomen CT revealed a lesion in the pancreas, and postoperative pathology also supported an “islet cell tumor” diagnosis. Hence, the patient was diagnosed with insulinoma. After tumor excision, he had no more hypoglycemia.

As another important etiology of EHH, TBIRS is a very rare autoimmune disorder characterized by circulating polyclonal autoantibodies against the insulin receptor [9, 10], disrupting glucose homeostasis. Typically, these insulin receptor antibodies are polyclonal IgG autoantibodies. They bind to the insulin receptor with high affinity, inevitably attenuating insulin action. This results in severe insulin resistance with compensated hyperinsulinemia. The plasma glucose profile can show persistent hyperglycemia, fasting hypoglycemia and postprandial hyperglycemia, or fasting and postprandial spontaneous hypoglycemia without hyperglycemia. TBIRS was first reported by Kahn et al. in 1976 [11] and occurs most in middle-aged black women. Its clinical manifestations include hyperinsulinemia, severe insulin resistance, hyperandrogenism, acanthosis nigricans, and hyperglycemia or hypoglycemia, depending on the blocking or stimulating activity of the antibodies [12]. It is generally believed that at high titers the antibody acts as an antagonist at the receptor, whereas at low titers it acts as a stimulatory agonist, resulting in different degrees of hyperglycemia and/or hypoglycemia [13]. The golden standard for diagnosing TBIRS is the detection of autoantibodies against the insulin receptor, but no commercial kit is available for automated biochemistry platforms, impairing the routine detection of those antibodies in the hospital [14]. The diagnosis of TBIRS relies more on clinical symptoms. TBIRS is usually complicated with other autoimmune diseases such as systemic lupus erythematosus, systemic sclerosis, and Sjogren’s syndrome [9, 10]. According to the literature, effective treatment of these underlying diseases is correlated with remission in TBIRS cases [15]. Hypoglycemia is an important cause of death for TBIRS [9, 16]. In the treatment of TBIRS, immunotherapy has shown more positive outcomes [10, 17]. In our study, case 2 presented recurrent fasting hypoglycemia, postprandial hyperglycemia, increased androgen levels, significant hyperinsulinemia, and relatively normal C-peptide levels. In addition, the patient had primary biliary cirrhosis and Sjogren’s syndrome. Although insulin receptor antibodies were not detected due to technical limitations, the patient should be considered as TBIRS according to her clinical characteristics and laboratory results. After treatment with glucocorticoids and antidiabetic agents, her glycemia became stable (5.3–12.8 mmol/L). No hypoglycemia recurred during 3 months of follow-up.

IAS is another common cause of EHH [18]—which also an autoimmune disease, like TBIRS—is characterized by hyperinsulinemic hypoglycemia associated with IAA. IAS is the result of genetic and environmental interactions, with common triggers including sulfhydryl compounds [19, 20]. Diagnosis of IAS must be based on high titers of IAA. Dramatically, when patients with IAS have spontaneous hypoglycemia, their serum insulin levels can be even higher than 1000 mIU/L [21, 22], markedly higher than the highest insulin levels observed in insulinoma patients, but the C-peptide levels are far lower than the insulin levels, showing the separation of C-peptide and insulin. IAS is a self-remitting disease with a good prognosis, which is usually relieved within 3 to 6 months after stopping drug treatment. In cases with troublesome and refractory hypoglycemia, corticosteroids may be necessary. In the present case study, shortly after taking sulfhydryl-containing medicine due to drug-induced liver injury, case 3 developed frequent hypoglycemia with inappropriately high insulin levels (>1000 mU/L). Further examination revealed very high serum IAA levels. The patient was definitively diagnosed with IAS. After treatment with glucocorticoid, he did not develop hypoglycemia.

In conclusion, effective treatment is dependent on proper diagnosis. In patients with EHH, we should primarily identify the etiology according to their clinical symptoms, blood glucose, serum insulin, C-peptide, relevant antibodies, and imaging examination, to provide effective treatment and eliminate hypoglycemia.
